# Treatment of COVID‐19 patients with the anti‐CD6 antibody itolizumab

**DOI:** 10.1002/cti2.1218

**Published:** 2020-11-25

**Authors:** Armando Caballero, Lázaro M Filgueira, Julio Betancourt, Naivy Sánchez, Carlos Hidalgo, Alberto Ramírez, Alejandro Martinez, Rolando E Despaigne, Alberto Escalona, Henrry Diaz, Elio Meriño, Lilia M Ortega, Ulises Castillo, Mayra Ramos, Danay Saavedra, Yanelda García, Geydi Lorenzo, Meylán Cepeda, Maylén Arencibia, Leticia Cabrera, Milagros Domecq, Daymys Estévez, Carmen Valenzuela, Patricia Lorenzo, Lizet Sánchez, Zaima Mazorra, Kalet León, Tania Crombet

**Affiliations:** ^1^ Intensive Care Unit Arnaldo Milián Castro University Hospital Santa Clara Cuba; ^2^ Intensive Care Unit Manuel “Piti” Fajardo Rivero Hospital Santa Clara Cuba; ^3^ Intensive Care Unit Salvador Allende Hospital Havana Cuba; ^4^ Intensive Care Unit Joaquín Castillo Hospital Havana Cuba; ^5^ Intensive Care Unit Faustino Pérez Hospital Matanzas Cuba; ^6^ Intensive Care Unit Frank País Hospital Havana Cuba; ^7^ Intensive Care Unit Pedro Kouri Institute Havana Cuba; ^8^ Emergency Department Ministry of Cuban Health Havana Cuba; ^9^ Clinical Research Direction Center of Molecular Immunology (CIM) Havana Cuba; ^10^ Research Direction Center of Molecular Immunology (CIM) Havana Cuba

**Keywords:** CD6, COVID‐19, cytokine release syndrome, itolizumab, monoclonal antibody, SARS‐CoV2

## Abstract

**Objectives:**

COVID‐19 can lead to a hyperinflammatory state. CD6 is a glycoprotein expressed on mature T lymphocytes which is a crucial regulator of the T‐cell activation. Itolizumab is a humanised antibody targeting CD6. Nonclinical and clinical data in autoimmune diseases indicate that it lowers multiple cytokines primarily involving the Th1/Th17 pathway. The primary objective of this study was to assess the impact of itolizumab in arresting the lung function deterioration of COVID‐19 patients. Secondary objectives included safety, duration of ventilation, 14‐day mortality and evaluation of interleukin 6 concentration.

**Methods:**

Patients with confirmed SARS‐CoV‐2 received itolizumab in combination with other therapies included in the national protocol for COVID‐19.

**Results:**

Seventy critical, severe or moderate patients were treated with itolizumab in 10 Cuban hospitals. Median age was 68, and 94% had comorbidities. After 72 h, most patients improved the PO_2_/FiO_2_ ratio and reduced FiO2 requirements. Ventilation time was 8 days for critical and 1 day for severe cases. Ten patients had related adverse events while 3 subjects developed related serious events. In 30 patients, interleukin 6 decreased in individuals with high level and did not change in those with lower concentration. Fourteen‐day lethality rate was 4% and 18% for moderate and severe patients, respectively. The proportion of moderate or severe patients with ventilation or death at day 14 was 9.8%. Time to treatment, neurological manifestations and biomarkers such as NLR were significantly associated with higher lethality.

**Conclusions:**

The opportune administration of itolizumab might interrupt the hyperinflammatory cascade and prevent COVID‐19 morbidity and mortality.

## Graphical abstract

In this study, 70 patients with confirmed SARS‐CoV‐2 received itolizumab in combination with other therapies included in the Cuban protocol for COVID‐19. The antibody reduced IL‐6 level or prevented its oversecretion. Most patients showed pulmonary improvement and were rapidly discharged from the ICU. We conclude that the timely use of itolizumab might interrupt the hyperinflammatory cascade and reduce COVID‐19 morbidity and mortality.

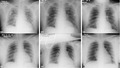

## Introduction

COVID‐19 is an acute respiratory disease caused by the highly pathogenic coronavirus SARS‐CoV‐2. The disease ranges from minimal symptoms to significant hypoxia with extrapulmonary involvement, which can be fatal.^1^ The viral infection can lead to a hyperinflammatory state known as cytokine release syndrome.^1^

In COVID‐19 autopsies, lympho‐monocytic infiltrates (CD3^+^ T cells over monocytes) have been found.^2^ Ackermann *et al*. described that the lungs from deceased patients with COVID‐19 or influenza has high infiltration of CD3^+^ T lymphocytes. CD4^+^ T cells were more abundant in lungs from COVID‐19 patients.^3^

CD6 is a glycoprotein expressed on the surface of mature T cells, B‐1 lymphocytes and immature B cells. CD6 is very important for the immunological synapse between the antigen‐presenting cells and the activated T lymphocytes.^4^ Interaction between CD6 and its main ligand, ALCAM (CD166), triggers cell proliferation and secretion of pro‐inflammatory cytokines.^5^

Although the exact mechanisms causing the exaggerated response against SARS‐CoV2 remain partially elusive,^6^ we hypothesise that targeting CD6 with an antagonistic antibody could dampen the systemic inflammation and reduce the morbidity and mortality associated with the disease.

Itolizumab is an IgG1 humanised monoclonal antibody (MAb) binding domain 1 of human CD6 that was generated at the Center of Molecular Immunology (Havana, Cuba).^7^ This MAb prevents the activation and proliferation of T cells. This inhibition leads to a marked reduction of pro‐inflammatory cytokines, involving the Th17 and Th1 pathway, including interleukin (IL) 17A, TNF‐α, IL‐6, interferon (IFN)‐γ and IL‐2. Itolizumab exhibits weak antibody‐dependent cellular cytotoxicity and does not induce apoptosis or complement‐dependent cytotoxicity.^8^ It has been widely used in patients with rheumatoid arthritis and psoriasis, significantly reducing serum levels of IL‐6, TNF‐α and IFN‐γ.^9^, ^10^ In a phase III clinical trial in patients with moderate‐to‐severe psoriasis conducted in India, itolizumab was effective and well tolerated.^11^ In total, 225 subjects were allocated to 2 different itolizumab arms (A or B) or placebo (C). Patients in arm A received 0.4 mg kg^−1^ of the antibody as induction while subjects in arm B were treated with 1.6 m kg^−1^. After 12 weeks, 27%, 36.4% vs. 2.3% of the patients treated in arm A, B or C had at least 75% improvement of Psoriasis Area and Severity Index. Acute infusion reactions were more frequent in the antibody than placebo arms (20.0%, 16.7% and 2.3% in A, B and C). Infections were more common in the placebo group.^11^

In view of the well‐proven inhibitory effect of itolizumab on T‐cell activation, an expanded‐access study was approved in Cuba. Here, we present the results of safety and clinical outcome of 70 critical, severe and moderate COVID‐19 patients treated with the antibody in 10 hospitals.

## Results

### Patient characteristics and treatment

Seventy SARS‐CoV2‐confirmed patients (39 male and 31 female) with moderate, severe or critical disease were treated with itolizumab in 10 Cuban hospitals from 4 April to 13 May 2020. Critical and severe patients received therapy at the intensive care unit (ICU). Twenty‐five patients had mechanical ventilation at the time of treatment. Moderate individuals received the antibody infusion at the hospital general wards. Table 1 shows demographics as well as comorbidities of the enrolled population. Overall, the mean and median age was 68 (28–100) and 94% had at least one comorbidity. Forty‐three patients (61.4%) had 2 or more associated diseases (Table 1). The most frequently associated concomitant conditions were hypertension (65.7%), cardiovascular disease (32.9%) and diabetes mellitus (31.4%). The median age of the severe and critical patients was 66 (29–92). Remarkably, the median age of the moderately ill patients was 75 and the majority (19 of 25, 76%) entered the study after a local transmission event in a nursing home. This elderly population had a high prevalence of dementia and nutrition deficit. Most patients were on treatment with lopinavir/ritonavir, chloroquine and IFNα2b before trial entry. Table 2 summarises the most important concomitant therapies after enrolment. Only 66% of the ICU patients received anticoagulants and 55% of the critically ill individuals continue using IFNα2b, in spite of disease worsening.

**Table 1:** Demographics and comorbidities of patients at baseline

Demographic	Critical		Severe		Moderate		Total	
	Freq.	%	Freq.	%	Freq.	%	Freq.	%
	29	100	16	100	25	100	70	100
Gender								
Female	11	37.9	12	75.0	16	64.0	39	55.7
Male	18	62.1	4	25.0	9	36.0	31	44.3
Skin colour								
White	19	65.5	9	56.3	15	60.0	43	61.4
Mixed	9	31.0	4	25.0	3	12.0	16	22.9
Black	1	3.4	3	18.8	4	16.0	8	11.4
ND					3	12.0	3	4.3
Age								
Mean ± SD	67.4 ± 14.0		66.9 ± 22.5		71.2 ± 17.7		68.7 ± 17.4	
Median ± IR	66.0 ± 26.0		81.5 ± 39.0		75.0 ± 23.0		68.0 ± 30.0	
min; max	(44; 92)		(29; 90)		(28; 100)		(28; 100)	
Patients with 1 comorbidity	29	100.0	16	100.0	21	84.0	66	94.3
Hypertension	20	69.0	10	62.5	16	64.0	46	65.7
Dementia	5	17.2	8	50.0	11	44.0	24	34.3
Cardiovascular diseases	11	37.9	4	25.0	8	32.0	23	32.9
Diabetes mellitus	12	41.4	4	25.0	6	24.0	22	31.4
Bronchial asthma	8	27.6	4	25.0	2	8.0	14	20.0
Nutrition deficit	1	3.4	1	6.3	10	40.0	12	17.1
Renal failure	6	20.7	3	18.8	0	0.0	9	12.9
COPD	4	13.8	–	–	5	20.0	9	12.9
Obesity	4	13.8	2	12.5	1	4.0	7	10.0
Smoker	1	3.4	3	18.8	2	8.0	6	8.6
Hypothyroidism	3	10.3	1	6.3	–	–	4	5.7
Cancer	4	13.8	–	–	–	–	4	5.7

**Table 2:** Most frequently used concomitant medications

	Critical ill		Severe ill		Moderate ill		Total	
	Freq.	%	Freq.	%	Freq.	%	Freq.	%
Lopinavir/ritonavir	29	100.0	16	100.0	23	100.0	68	100.0
Chloroquine	26	89.7	15	93.8	22	95.7	63	92.6
Antibiotics	29	100.0	16	100.0	7	30.4	52	76.5
Low molecular weight heparin (LMWH)	19	65.5	11	68.8	21	91.3	51	75.0
Interferon α2B	16	55.2	7	43.8	14	60.9	37	54.4

The time lag between first symptoms and itolizumab was 10 days for critical, 7.5 days for severe and 1 day for moderately ill subjects. All patients completed the first itolizumab infusion, 41 (58.8%) received 2 doses while 3 patients (4.3%) had 3 doses. The median time between the first and second dose was 3 days, while the median interval between the second and third dose was 5 days.

### Efficacy evaluation

**Figure 1:**
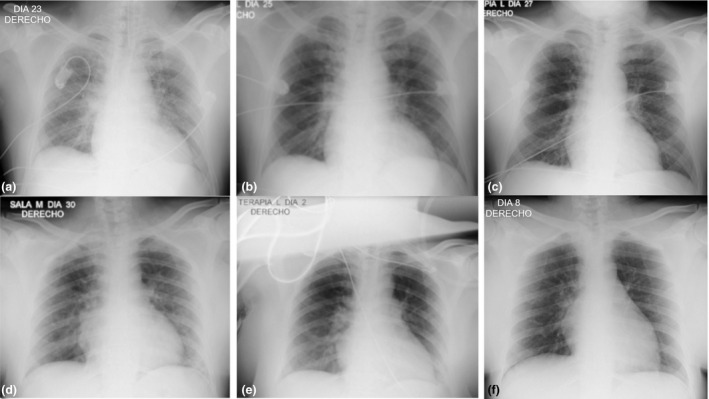
Serial chest radiographs showed significant recovery in 2 COVID‐19 patients after treatment with itolizumab. Patient 1: **(a)** (Before itolizumab): bilateral hilar vascular thickening, greater on the right side. Right para‐cardiac shadow opacity. **(b)** (48 h after itolizumab): decreased bilateral hilar vascular thickening. Decreased opacity in the right para‐cardiac shadow and thickening of the right basal hilum. No pleuro‐pulmonary lesions. **(c)** (5 days after itolizumab): favorable radiological evolution, with disappearance of bilateral hilar vascular thickening and the right para‐cardiac opacity. No pleuro‐pulmonary lesions. Patient 2: **(d)** (Before itolizumab): diffuse veil opacities that project in the right para‐cardiac region and in the lower left lung field. **(e)** (48 h after itolizumab): decrease in veil opacities. **(f)** (8 days after itolizumab): no pleuro‐pulmonary lesions. Favorable radiological evolution, with disappearance of the lung lesions.

After 72 h, 83.3% of the severe and 63.6% of the critical patients did not need FiO_2_ intensification to keep oxygen saturation. Furthermore, 83.3% and 55% of the severe and critical patients significantly improved the PaO_2_/FiO_2_ index (ratio of arterial partial pressure of oxygen to fraction of inspired oxygen). Three days postitolizumab infusion, 60% and 15% of the severe and critical patients, respectively, had radiological improvement of the multifocal interstitial pneumonia. Figure 1 displays chest X‐rays from 2 representative patients showing prompt radiological recovery after the antibody administration.

Only 7 patients (3 critical and 4 severe) of the 45 nonventilated at trial entry required further mechanical ventilation. Median time to ventilator weaning was 8 days for critical and 1 day for severe cases, after itolizumab. Four of the 25 moderately ill patients (16%) required ICU admission after itolizumab. Three of these four individuals successfully recovered. At day 14, 23 of the 25 moderately ill patients (92%) were discharged from hospital.

Overall, 24 patients (1 moderate, 3 severe and 20 critical) died by day 14. The 14‐day lethality rate was 4%, 18.7% and 69% for moderate, severe and critical individuals. For moderate and severe patients combined, the proportion of patients with noninvasive ventilation, intubation or death at day 14 was 9.8%.

### Cytokine evaluation

**Figure 2:**
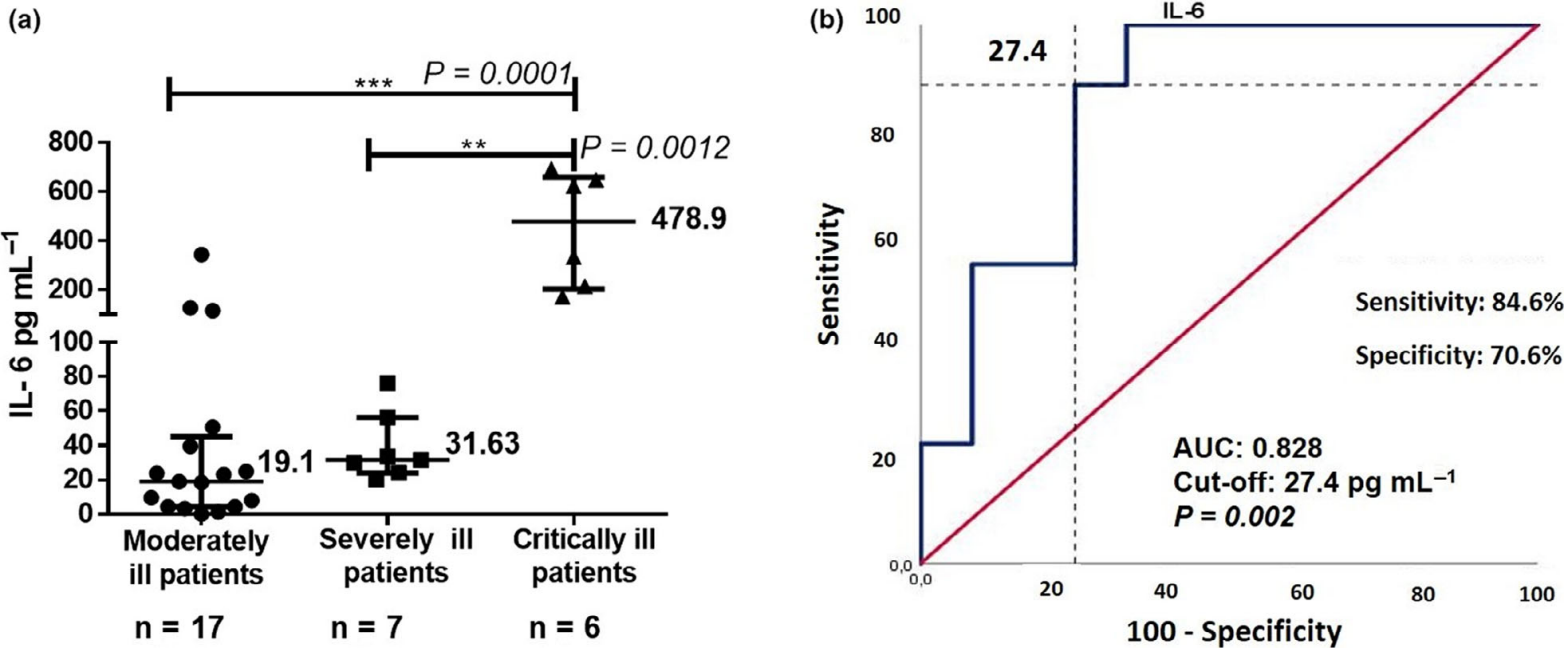
IL‐6 serum concentration in COVID‐19 patients before and 48 hours after treatment with itolizumab. **(a)** Median IL‐6 levels in the 3 groups of COVID‐19 patients. All experiments were performed in duplicate (Human IL‐6 Quantikine ELISA Kit). **(b)** ROC curves of IL‐6 predictive value for COVID‐19 severity. The asterisks indicate statistically significant differences among the groups (**P < 0.001, ***P < 0.0001) using the Mann–Whitney test. ROC, receiver operator characteristic; AUC, area under the curve.

In 30 patients, serum concentration of interleukin 6 (IL‐6) was determined at trial entry. IL‐6 level was significantly higher in critical as compared to severe or moderate patients. Median IL‐6 concentration was 478.5 pg mL^−1^, 31.6 pg mL^−1^ and 19.1 pg mL^−1^ for critical, severe and moderate individuals, respectively (Figure 2). A receiver operating characteristic (ROC) curve was drawn to define the IL‐6 level that predicts severity (Figure 2). In our dataset, the IL‐6 concentration associated with gravity was 27.4 pg mL^−1^. A short kinetic of serum IL‐6 was measured before and 48 h after itolizumab in 23 patients with samples. Patients were grouped considering the predetermined cut‐off for disease severity: thirteen patients had levels above 27.4 pg mL^−1^ while 10 patients had lower IL‐6 levels. IL‐6 significantly decreased for individuals with concentration higher than 27.4 pg mL^−1^ (Wilcoxon *P* = 0.028): median IL‐6 level was 116.3 and 78.8 pg mL^−1^, before and after itolizumab. In contrast, the median IL‐6 level for subjects below the cut‐off was 13.8 pg mL^−1^. The median post‐treatment IL‐6 level in the ‘low inflamed’ group was 15.9 pg mL^−1^. Median pre‐ and postitolizumab levels were not significantly different. We concluded that after itolizumab, IL‐6 decreased in patients with high inflammation and did not significantly change in subjects with low levels. The cytokine release syndrome in the ‘low inflamed’ patients was not triggered following MAb infusion. IL‐1 and TNF‐α were undetectable in the same samples.

### Safety

Overall, 22 of 70 patients (31.4%) had adverse events (related or unrelated), while only 10 individuals (14.3%) developed related adverse events. In all, 26 adverse events out of 84 (31%) were classified as possibly or probably associated with itolizumab. None of the adverse events was definitely attributed to the antibody. Most frequently related adverse events were mild‐to‐moderate chills (5 patients), hypotension (5 patients), fever (3 patients), tachycardia (2 patients) and hypoxia (2 patients). Three patients (4.3%) had related serious adverse events (SAE). SAE consisted of airways hyper‐reactivity, fever, hypotension, rash, chills, hypoxia, shock and cyanosis. SAE were classified as possibly related with itolizumab. The hypoxia and shock led to the death of one patient, while the remaining SAE were controlled after decreasing the infusion rate or with antihistaminics. The 3 patients presenting with SAE were on mechanical ventilation and receiving lopinavir/ritonavir, chloroquine and IFNα2b at the moment of itolizumab infusion.

One death was classified as possibly related with the antibody. This patient was a 62‐year‐old female with morbid obesity and type 2 diabetes. Her first respiratory symptoms started 12 days before trial enrolment. Upon COVID‐19 confirmation, she had diffused inflammatory lesions in both pulmonary fields and hypoxaemia (PO2/FiO2 = 109). At hospital admission, this patient required invasive ventilation including alveolar recruitment manoeuvres (PEEP = 15). In addition to lopinavir/ritonavir, chloroquine and IFNα2b, she was receiving meropenem and vancomycin. This patient received itolizumab after 5 days on invasive mechanical ventilation. Before the antibody administration, she also had hypernatraemia, hyperglycaemia and persistent fever of 39–40°C for 48 h. She died on the same day of the antibody infusion. The cause of death was multiple organ dysfunction. Considering the patient’s comorbidities together with her clinical and radiological conditions at the moment of itolizumab infusion, death was likely to be associated with the natural course of the disease.

In our series, the main cause of death was multiple organ dysfunction, pulmonary thrombosis or thromboembolic events. Secondary infections were not exacerbated after the antibody.

### Biomarker evaluation

Neutrophil, lymphocyte and platelet count was evaluated before itolizumab and then daily for a week. At inclusion, neutrophil count correlated with severity: moderate (3.7 × 10^9^ L^−1^), severe (5.4 × 10^9^ L^−1^) and critical (10.8 × 10^9^ L^−1^). Neutrophil count did not significantly augment in the 7 days following the anti‐CD6 MAb. At trial admission, the rate of patients with grade 1 lymphopenia (< 0.8 × 10^9^ L^−1^) was 13% for moderate and severe vs. 43% of critical patients. A week after itolizumab, the proportion of subjects with grade 2 lymphopenia (< 0.5 × 10^9^ L^−1^) was 0, 11% and 20% for moderate, severe and critical individuals. Platelet count was within the normal range for the three severity groups before and after treatment.

Receiver operating characteristic analysis was employed to define predictive cut‐offs for severity and mortality of several laboratory biomarkers. Table 3 shows the predictive value of triglycerides, AST, D‐dimer, IL‐6, absolute leucocyte count (ALC), neutrophils, neutrophil‐to‐lymphocyte ratio (NLR) and platelet‐to‐lymphocyte ratio (PLR) on COVID‐19 severity or mortality. In addition, the odds ratios of death for several independent variables including demographics, important comorbidities as well as laboratory parameters were estimated (Table 4). Using univariate logistic regression analysis, time between symptoms and itolizumab, the onset of neurological manifestations, AST, D‐dimer, ALC, neutrophil count, NLR, PLR and IL‐6 were shown to be significantly associated with higher lethality risk. The only comorbidity associated with a significantly higher death chance was chronic kidney disease. The biomarker associated with the highest lethality risk was NLR (OR 26.44 [95% CI 5.78; 120.8]). Elevated AST, neutrophil count and IL‐6 were the next biomarkers associated with significantly increased mortality risk.

**Table 3:** Predictive values of triglycerides, aspartate aminotransferase (AST), D‐dimer, interleukin 6 (IL‐6), absolute leucocyte count (ALC), neutrophils, neutrophil‐to‐lymphocyte ratio (NLR) and platelet‐to‐lymphocyte ratio (PLR) associated with COVID‐19 severity or mortality according to ROC analysis

	Area	Sig.	95% CI		Sensitivity	Specificity	Cut‐off
Severity							
Triglycerides	0.756	0.003	0.617	0.896	78.6%	65%	1.24 mmol L^−1^
AST	0.858	0.000	0.749	0.966	82.8%	85%	20.5 IU L^−1^
D‐Dimer	0.783	0.009	0.603	0.964	80%	78.6%	1.35 µg mL^−1^
IL‐6	0.828	0.002	0.683	0.973	71.4%	73.9%	27.4 pg mL^−1^
ALC	0.838	0.000	0.740	0.936	82.9%	70.8%	6.55 × 10^9^ L^−1^
Neutrophils	0.840	0.000	0.735	0.945	94.7%	70.8%	4.34 × 10^9^ L^−1^
NLR	0.799	0.000	0.685	0.913	70.6%	82.6%	4.91
PLR	0.673	0.029	0.524	0.823	75.8%	69.6%	135.0
Mortality							
AST	0.802	0.000	0.667	0.937	83.3%	71%	22.1 IU L^−1^
D‐Dimer	0.742	0.035	0.515	0.969	80%	63.2%	1.35 µg mL^−1^
IL‐6	0.770	0.033	0.527	1.000	71.4%	73.9%	53.4 pg mL^−1^
ALC	0.727	0.003	0.592	0.863	72.7%	65.1%	7.60 × 10^9^ L^−1^
Neutrophils	0.765	0.001	0.636	0.895	81.0%	65.9%	5.57 × 10^9^ L^−1^
NLR	0.894	0.000	0.804	0.984	82.4%	85.0%	8.85
PLR	0.711	0.014	0.556	0.866	81.3%	60%	146.2

**Table 4:** Univariate logistic regression analysis. The highlighted variables are significantly associated with higher odds of death.

		Death Odds ratio	IC 95%	
**Generals**	Age (> 65)	1.680	0.601	4.697
	**Time between symptoms and itolizumab (> 7)**	**5.625**	**1.862**	**16.989**
	**Neurological symptoms**	**4.778**	**1.076**	**21.224**
Comorbidities	Hypertension	0.613	0.220	1.709
	Diabetes Mellitus	2.024	0.712	5.753
	Cardiovascular disease	1.813	0.644	5.102
	COPD	0.952	0.216	4.197
	Cancer	2.000	0.264	15.163
	**Chronic renal disease**	**4.778**	**1.076**	**21.224**
	Asthma	1.583	0.478	5.246
	Obesity	1.500	0.307	7.326
	Nutrition deficit	0.327	0.066	1.634
**Baseline lab biomarkers**	**AST (> 22.1 IU L^−1^)**	**10.500**	**2.462**	**44.78**
	**D‐dimer (> 1.35 µg mL^−1^)**	**6.857**	**1.124**	**41.827**
	**ALC (> 7.60 × 10^9^ L^−1^)**	**4.978**	**1.610**	**15.387**
	**Neutrophils (> 5.57 × 10^9^ L^−1^)**	**8.196**	**2.311**	**29.073**
	**NLR (> 8.85)**	**26.444**	**5.788**	**120.819**
	**PLR (> 146.2)**	**6.500**	**1.594**	**26.511**
	**IL‐6 (> 53.4 pg mL^−1^)**	**7.083**	**1.075**	**46.478**

The highlighted variables are significantly associated with higher odds of death.

## Discussion

Cytokine release syndromes encompass a group of disorders with multiple inflammatory aetiologies that result in overwhelming inflammation, multiple organ dysfunction or even death.^12^

The role of activated T cells in the pathogeny of COVID‐19 is not fully understood. It has been proposed that antigen‐presenting cells secrete level‐1 cytokines (IL‐1, TNF‐α, IL‐6) that activate T cells. Upon activation, T lymphocytes secrete further cytokines designed as level 2 cytokines (IFN‐γ, IL‐21, IL‐17) that boost the innate and adaptive immune response against the virus.^13^

CD6, a member of the scavenger receptor cysteine‐rich superfamily, has been considered a crucial regulator of T‐cell activation.^14^ Itolizumab is a nondepleting antibody that targets CD6.^8^ Nonclinical and clinical data indicate that the antibody acts directly on T cells and lowers multiple cytokines and signal transduction factors primarily involving the Th1 and Th17 pathway.^8^

We hypothesise that using an anti‐CD6 antibody in COVID‐19 patients could reduce the concentration of several pro‐inflammatory cytokines, including IL‐6, IFN‐γ and IL‐17, representing an advantage as compared to single‐cytokine targeting antibodies. Itolizumab administration can also favor regulation instead a Th1 fate.^15^

The aim of the trial was to control the overwhelming systemic inflammation or to prevent the cytokine release in 70 COVID‐19 patients. IL‐6 is considered the main driver of the hyperinflammatory syndrome in COVID‐19.^1^ Elevated levels of IL‐6 were found in more than half of the sick patients and are associated with respiratory failure and mortality.^16^ Itolizumab successfully reduced this cytokine in individuals with very high concentration and prevented IL‐6 boost in moderate patients at high risk of aggravation. We conclude that the antibody inhibited T‐cell activation and hindered cytokine secretion. It is documented that IL‐6 ablation may depress B‐cell response and delay antiviral response.^17^ In our series, the viral load was negative in all clinically recovered patients, suggesting that itolizumab did not hamper the adaptive immune response. IL‐1 and TNF‐α were not quantifiable at the same time points. TNF‐α and IL‐1 are the first cytokines to be released in sepsis, triggering the secretion of IL‐6.^18^

Regarding safety, 14.3% of the treated patients had related adverse events while 4.2% had possibly related SAE. Remarkably, no important changes in lymphocyte count beyond the natural course of the disease were seen.^19^ Bacterial or fungal infections did not increase after the antibody. Most common adverse events consisted of infusion reactions and were similar to those previously found in autoimmune diseases.^8^, ^11^ In Cuban patients with rheumatoid arthritis or psoriasis, the most frequent adverse events were chills and fever, followed by nausea, vomiting, headache, skin rash and tremors.^8^, ^18^ In a phase III trial done in India, infusion reactions occurred in 20% of the psoriatic patients. These mild‐to‐moderate events were more frequent after the first antibody infusion.^11^ SAE were reported in four patients.^11^

Our series had a very poor prognosis: 37% were older than 80% and 94% had important associated conditions. It is well accepted that comorbidities and age exacerbate the disease manifestations,^17^ increasing the susceptibility of endothelial damage and dysregulation of the metabolic syndrome.^20^ After combining itolizumab with the standard of care, the 14‐day lethality rate was 4% for moderate and 18% for severe patients. Moreover, for moderate and severe patients combined, the proportion of patients with noninvasive ventilation, intubation or death at day 14 was 9.8%. This rate compares favorably with a recent report of the use of tocilizumab or placebo in 130 patients with moderate or severe COVID‐19 in France. In a multicentre, randomised clinical trial, the proportion of patients with noninvasive ventilation, intubation or death at day 14 was 36% with usual care and 24% with tocilizumab.^21^

In a separate randomised trial enrolling 243 moderate patients in the United States, 18% of the patients in the tocilizumab group and 14.9% of the patients in the placebo group had worsening of disease at 14 days. After itolizumab, 8% of the moderate patients had disease worsening at day 14.^22^ In a case–control study, Díaz *et al*.^23^ found that for every 3 moderately ill patients treated with itolizumab, one admission into the ICU was prevented. Additionally, treatment with itolizumab reduced 10 times the risk of death as compared with the control group.^23^

Lung function improved in severe and critical patients, when considering FiO_2_ reduction, oxygenation index or chest imaging. In spite of the improvement of the respiratory distress, the survival rate was 31% in critical patients. Low survival could be associated with the lack of anticoagulation and the prolonged use of IFNα2b, beyond disease progression. Delayed type I IFN might have further activated the innate immune system,^24^, ^25^ precluding a higher impact of the antibody. In addition, many critical patients were enrolled after 72 h of mechanical ventilation.

Other anti‐inflammatory drugs have been evaluated to control the cytokine storm associated with COVID‐19. In the largest randomised clinical trial, dexamethasone reduced the 28‐day mortality in patients with invasive mechanical ventilation or oxygen alone.^26^ However, mortality did not decrease in patients without supplemental oxygen.^26^ The larger effect was seen in ventilated patients (29.3% vs. 41.4%) while the effect was smaller in subjects receiving oxygen without invasive mechanical ventilation (23.3% vs. 26.2%).^26^ In contrast, there was no clear effect of dexamethasone among patients who were not receiving any respiratory support at randomisation (17.8% vs. 14%).^26^

Overall, we conclude that itolizumab may be better used before patients reach critical illness or at the onset of the severe respiratory distress. In critical patients, the consequences of hyperinflammation including micro or macrovascular thrombosis, hyaline membrane and alveolar injury might be irreparable.^6^ Systemic inflammation might have also irreversibly affected the glomeruli, heart or liver.^3^

Since the opportune use of itolizumab is crucial, it is necessary to identify those patients most likely to progress to a severe form of the disease. Biomarkers can assist in predicting severity and lethality. In our study, the biomarkers associated with the greatest mortality risk were elevated NLR, AST, neutrophil count and IL‐6. Other markers previously found to be useful for COVID‐19 risk stratification,^27^, ^28^ such as C‐reactive protein, ferritin, lactate dehydrogenase and procalcitonin, were not evaluated in all patients. NLR, a well‐established systemic inflammatory marker,^29^ was associated with the highest lethality.

Even though this is the largest, multicentric study done so far on the effect of itolizumab on COVID‐19 patients, results should be interpreted with caution. The study was not randomised and patients were not followed after 14 days. Interleukin 6 and other inflammatory cytokines were evaluated before and only 48 h after the MAb infusion. In addition, since patients were enrolled at the beginning of the pandemic in the country, the standard of care used together with the antibody was not optimal, according to the current state of the art. Finally, many patients were treated with itolizumab very late in the natural course of illness.

In summary, itolizumab reduced IL‐6 level or blocked its oversecretion. Most patients showed pulmonary improvement and were rapidly discharged from the ICU. For moderate and severe patients, worsening and lethality rate is encouraging when comparing with the standard of care or other immunomodulatory drugs. A randomised trial of 30 patients with moderate and severe COVID‐19 recently concluded in India.^15^ According to the initial data, none of the 20 patients assigned to itolizumab died, while 3 of the 10 individuals given usual care died.^30^ While new trials are available, we speculate that the timely administration of itolizumab, guided by biomarkers, can interrupt the hyperinflammatory cascade and might prevent morbidity and mortality related to the cytokine release syndrome. The safety and effectiveness of itolizumab in COVID‐19 will be evaluated in a global, multinational phase III, randomised clinical trial (ClinicalTrials.gov number: NCT04605926).

## Methods

### Study design

This study was an open‐label, expanded‐access trial in which moderate, severe or critical SARS‐CoV‐2 Cuban patients received itolizumab in combination with other therapies included in the national protocol for COVID‐19. Diagnose was confirmed by reverse transcription–polymerase chain reaction (RT‐PCR). Other therapies consisted in lopinavir/ritonavir, chloroquine and recombinant IFNα2b. Some patients also received low molecular weight heparin (LMWH) since anticoagulants were included in the SARS‐CoV2 national protocol, after trial initiation. Inclusion criteria were as follows: age ≥ 18 years, confirmed multifocal interstitial pneumonia, need for oxygen therapy to maintain saturation (SaO_2_) > 93% and worsening of lung involvement. In addition, subjects with radiological evidence of lower respiratory disease but without oxygen therapy were enrolled if they have one of the following conditions: wheezing or irregular speech, respiratory frequency greater than 22 breaths per minute, PaO_2_ < 65 mmHg, persistent fever ≥ 38°C for 48 h, decrease of haemoglobin, platelets or leucocytes below 9.2 g dL^−1^, 110 × 10^9^ L^−1^ or 5 × 10^9^ L^−1^, respectively, as compared to the original hospitalisation day for COVID‐19, triglycerides > 3 mmol L^−1^, increase in ferritin or absolute ferritin value ≥ 2000 ng mL^−1^, aspartate aminotransferase (AST) ≥ 30 IU L^−1^, D‐dimer increase, fibrinogen < 2.5 g L^−1^ or onset of neurological manifestations. Patients were excluded if they were receiving other biologics or had a known allergy to antibodies or any component of the formulation. Pregnant or breastfeeding females were also excluded.

The protocol was approved by the Ethics Committee of the Institute of Tropical Medicine ‘Pedro Kourí’ and by the Cuban Regulatory Agency. All investigations were conducted in accordance with the Helsinki Declaration. Written informed consent was obtained from all patients with full mental competence prior to itolizumab treatment. A legal guardian provided consent for patients with cognitive disorders. Additionally, consent was requested from the surviving ventilated patients, as soon as they recovered. The protocol was registered in the National Registry for Clinical Trials (http://rpcec.sld.cu/trials/RPCEC00000311‐En).

Critically ill patients were defined as those requiring mechanical ventilation or those who have respiratory failure (PaO_2_/FiO_2_) < 200, septic shock or multiple organ dysfunction. Severely ill individuals were those who have respiratory frequency > 30 breaths per min, SaO_2_ ≤ 93%, PaO_2_/FiO_2_ < 300 or lung infiltrates > 50%. Moderately ill patients have clinical and imaging evidence of lower respiratory disease and SaO_2_ > 93%. Only moderately ill patients with high risk of aggravation were included in the trial. Risk factors for worsening were age ≥ 65 and comorbidities associated with COVID‐19 mortality: hypertension, cardiovascular disease, diabetes mellitus, chronic kidney disease, cancer, chronic obstructive pulmonary disease (COPD), obesity and nutrition deficit.

The primary objective of the trial was to assess the impact of itolizumab in arresting the deterioration of lung function, measured as the proportion of patients without need to increase FiO_2_ to keep SaO_2_ above 93% and the rate of patients improving PaO_2_/FiO_2_, after 3 and 7 days of the antibody infusion. Other secondary objectives were the following: rate of patients who need mechanical ventilation, duration of ventilation and mortality rate after 14 days of the antibody administration.

Itolizumab‐related adverse events were reported and classified according to the Common Terminology Criteria for Adverse Events, version 5. Haemoglobin and complete blood count were done at baseline and then daily, up to 168 h of itolizumab administration. C‐reactive protein, triglycerides, fibrinogen, ferritin, AST and D‐dimer were evaluated at the same frequency. In 30 patients, IL‐6 was quantified before and at 48 h after itolizumab in serum. IL‐6 concentration was measured using a Quantikine ELISA Kit (S6050) from R&D Systems (Minneapolis, USA). IL‐1 and TNF‐α were evaluated with the human IL‐1 beta/IL‐1F2 Quantikine ELISA Kit (SLB50) Systems (Minneapolis, USA) or the human TNF‐α, Quantikine ELISA Kit (STA00D) (Minneapolis, USA).

Treatment consisted in one intravenous infusion of 200 mg of itolizumab diluted in 200 mL of sodium chloride (0.9%). Patients could receive a second dose of the antibody if they still had signs of respiratory distress. Infusion duration was at least 2 h. The research product was stored at 2–8°C. Itolizumab was formulated at a concentration of 5 mg mL^−1^ (25 mg per vial) in sterile buffer.

### Statistics

Demographic and clinical characteristics were reported according the disease severity groups. Categorical variables were displayed using descriptive statistics while metric variables were presented using the central tendency magnitude and dispersion: mean ± SD for variables with normal distribution or median and interquartile range for non‐normal data. The discriminative power of several biomarkers regarding disease severity and mortality was evaluated by receiver operating characteristic (ROC) analyses. The mortality odds ratio for several independent variables including demographics, comorbidities and laboratory parameters above the predetermined threshold for higher death risk were estimated. Analyses were made with the SPSS version 25 and R software.

## Conflict of interest

Fifteen authors currently work for the Center of Molecular Immunology, the institution that generated and originally patented itolizumab. The other authors do not have any commercial or financial relationships that could be taken as a potential conflict of interest.

## Author contributions

**Armando Caballero:** Funding acquisition; Investigation; Supervision. **Lazaro Filgueira:** Formal analysis; Investigation. **Julio Betancourt:** Formal analysis; Investigation. **Naivy Sánchez:** Investigation. **Carlos Hidalgo:** Formal analysis; Investigation. **Alberto D Ramírez:** Investigation. **Alejandro Martinez:** Investigation. **Rolando E Despaigne:** Investigation. **Alberto Escalona:** Investigation. **Elio Meriño:** Investigation. **Liliam Ortega‐Gonzalez:** Investigation. **Henrry Díaz:** Investigation. **Ulises Castillo:** Investigation. **Mayra Ramos:** Conceptualization; Formal analysis; Investigation. **Danay Saavedra:** Conceptualization; Formal analysis; Investigation; Writing‐original draft. **Yanelda Garcia:** Conceptualization; Investigation. **Geydi Lorenzo:** Data curation; Investigation. **Meylan Cepeda:** Data curation; Investigation. **Maylen Arencibia:** Data curation; Investigation. **Leticia Cabrera:** Data curation; Investigation. **Milagros Domecq:** Data curation; Investigation. **Daymys Estevez:** Investigation. **Carmen Valenzuela:** Formal analysis; Methodology; Writing‐original draft. **Patricia Lorenzo‐Luaces:** Formal analysis; Methodology; Writing‐original draft. **Lizet Sánchez:** Formal analysis; Methodology. **Zaima Mazorra:** Conceptualization; Formal analysis; Investigation. **Kalet Leon:** Formal analysis; Supervision. **Tania Crombet:** Conceptualization; Formal analysis; Investigation; Supervision; Writing‐original draft; Writing‐review & editing.

## References

[1] Atal S , Fatima Z . IL‐6 inhibitors in the treatment of serious COVID‐19: a promising therapy? Pharmaceut Med 2020; 34: 223–231. 32535732 10.1007/s40290-020-00342-z PMC7292936

[2] Aguiar D , Lobrinus JA , Schibler M , Fracasso T , Lardi C . Inside the lungs of COVID‐19 disease. Int J Legal Med 2020; 134: 1271–1274. 32458044 10.1007/s00414-020-02318-9 PMC7248187

[3] Ackermann M , Verleden SE , Kuehnel M *et al* Pulmonary vascular endothelialitis, thrombosis, and angiogenesis in Covid‐19. N Engl J Med 2020; 383: 120–128. 32437596 10.1056/NEJMoa2015432 PMC7412750

[4] Santos RF , Oliveira L , Carmo AM . Tuning T cell activation: the function of CD6 at the immunological synapse and in T cell responses. Curr Drug Targets 2016; 17: 630–639. 26028048 10.2174/1389450116666150531152439

[5] Dogra S , Uprety S , Suresh SH . Itolizumab, a novel anti‐CD6 monoclonal antibody: a safe and efficacious biologic agent for management of psoriasis. Expert Opin Biol Ther 2017; 17: 395–402. 28064543 10.1080/14712598.2017.1279601

[6] Lippi G , Sanchis‐Gomar F , Henry BM . COVID‐19: unravelling the clinical progression of nature's virtually perfect biological weapon. Ann Transl Med 2020; 8: 693. 32617313 10.21037/atm-20-3989 PMC7327324

[7] Osorio LM , Garcia CA , Jondal M , Chow SC . The anti‐CD6 mAb, IOR‐T1, defined a new epitope on the human CD6 molecule that induces greater responsiveness in T cell receptor/CD3‐mediated T cell proliferation. Cell Immunol 1994; 154: 123–133. 7509726 10.1006/cimm.1994.1062

[8] Hernandez P , Moreno E , Aira LE , Rodriguez PC . Therapeutic targeting of CD6 in autoimmune diseases: a review of cuban clinical studies with the antibodies IOR‐T1 and itolizumab. Curr Drug Targets 2016; 17: 666–677. 26844560 10.2174/1389450117666160201114308

[9] Aira LE , Hernandez P , Prada D *et al* Immunological evaluation of rheumatoid arthritis patients treated with itolizumab. mAbs 2016; 8: 187–195. 26466969 10.1080/19420862.2015.1105416 PMC4966522

[10] Aira LE , Lopez‐Requena A , Fuentes D *et al* Immunological and histological evaluation of clinical samples from psoriasis patients treated with anti‐CD6 itolizumab. mAbs 2014; 6: 783–793. 24594862 10.4161/mabs.28376 PMC4011922

[11] Krupashankar D , Dogra S , Kura M *et al* Efficacy and safety of itolizumab, a novel anti‐CD6 monoclonal antibody, in patients with moderate to severe chronic plaque psoriasis: results of a double‐blind, randomized, placebo‐controlled, phase‐III study. J Am Acad Dermatol 2014; 71: 484–492. 24703722 10.1016/j.jaad.2014.01.897

[12] Miossec P . Understanding the cytokine storm during COVID‐19: Contribution of preexisting chronic inflammation. Eur J Rheumatol 2020; 7: S97–S98. 32412405 10.5152/eurjrheum.2020.2062 PMC7431332

[13] Ghnewa YG , Fish M , Jennings A , Carter MJ , Shankar‐Hari M . Goodbye SIRS? Innate, trained and adaptive immunity and pathogenesis of organ dysfunction. Med Klin Intensivmed Notfmed 2020; 115: 10–14. 32291506 10.1007/s00063-020-00683-2

[14] Ma C , Wu W , Lin R *et al* Critical role of CD6^high^CD4^+^ T cells in driving Th1/Th17 cell immune responses and mucosal inflammation in IBD. J Crohns Colitis 2019; 13: 510–524. 30395204 10.1093/ecco-jcc/jjy179

[15] Loganathan S , Athalye SN , Joshi SR . Itolizumab, an anti‐CD6 monoclonal antibody, as a potential treatment for COVID‐19 complications. Expert Opin Biol Ther 2020; 20: 1025–1031. 32700604 10.1080/14712598.2020.1798399

[16] Grifoni E , Valoriani A , Cei F *et al* Interleukin‐6 as prognosticator in patients with COVID‐19. J Infect 2020; 81: 452–482. 10.1016/j.jinf.2020.06.008 PMC7278637 32526326

[17] Gubernatorova EO , Gorshkova EA , Polinova AI , Drutskaya MS . IL‐6: relevance for immunopathology of SARS‐CoV‐2. Cytokine Growth Factor Rev 2020; 53: 13–24. 32475759 10.1016/j.cytogfr.2020.05.009 PMC7237916

[18] Steeland S , Libert C , Vandenbroucke RE . A new venue of TNF targeting. Int J Mol Sci 2018; 19: 1442. 10.3390/ijms19051442 PMC5983675 29751683

[19] He R , Lu Z , Zhang L *et al* The clinical course and its correlated immune status in COVID‐19 pneumonia. J Clin Virol 2020; 127: 104361. 32344320 10.1016/j.jcv.2020.104361 PMC7152870

[20] Poor HD , Ventetuolo CE , Tolbert T *et al* COVID‐19 critical illness pathophysiology driven by diffuse pulmonary thrombi and pulmonary endothelial dysfunction responsive to thrombolysis. Clin Transl Med 2020; 13: e44. 10.1002/ctm2.44 PMC7288983 32508062

[21] Hermine O , Mariette X , Tharaux PL *et al* Effect of tocilizumab vs usual care in adults hospitalized with COVID‐19 and moderate or severe pneumonia: a randomized clinical trial. JAMA Intern Med 2020; e206820 10.1001/jamainternmed.2020.6820 PMC7577198 33080017

[22] Stone JH , Frigault MJ , Serling‐Boyd NJ *et al* Efficacy of tocilizumab in patients hospitalized with covid‐19. N Engl J Med 2020 10.1056/NEJMoa2028836 PMC7646626 33085857

[23] Diaz Y , Ramos‐Suzarte M , Martin Y *et al* Use of a humanized anti‐CD6 monoclonal antibody (itolizumab) in elderly patients with moderate COVID‐19. Gerontology 2020; 1–9. 10.1159/000512210 PMC7649683 33105142

[24] Channappanavar R , Fehr AR , Vijay R *et al* Dysregulated type I interferon and inflammatory monocyte‐macrophage responses cause lethal pneumonia in SARS‐CoV‐infected mice. Cell Host Microbe 2016; 19: 181–193. 26867177 10.1016/j.chom.2016.01.007 PMC4752723

[25] Channappanavar R , Fehr AR , Zheng J *et al* IFN‐I response timing relative to virus replication determines MERS coronavirus infection outcomes. J Clin Invest 2019; 129: 3625–3639. 31355779 10.1172/JCI126363 PMC6715373

[26] RECOVERY Collaborative Group , Horby P , Lim WS *et al* Dexamethasone in hospitalized patients with Covid‐19 –preliminary report. N Engl J Med 2020 10.1056/NEJMoa2021436 PMC7383595 32678530

[27] Henry BM , de Oliveira MHS , Benoit S , Plebani M , Lippi G . Hematologic, biochemical and immune biomarker abnormalities associated with severe illness and mortality in coronavirus disease 2019 (COVID‐19): a meta‐analysis. Clin Chem Lab Med 2020; 58: 1021–1028. 32286245 10.1515/cclm-2020-0369

[28] Ponti G , Maccaferri M , Ruini C , Tomasi A , Ozben T . Biomarkers associated with COVID‐19 disease progression. Crit Rev Clin Lab Sci 2020; 57: 389–399. 32503382 10.1080/10408363.2020.1770685 PMC7284147

[29] Dolan RD , Lim J , McSorley ST , Horgan PG , McMillan DC . The role of the systemic inflammatory response in predicting outcomes in patients with operable cancer: Systematic review and meta‐analysis. Sci Rep 2017; 7: 16717. 29196718 10.1038/s41598-017-16955-5 PMC5711862

[30] Atal S , Fatima Z , Balakrishnan S . Approval of Itolizumab for COVID‐19: A premature decision or need of the hour? BioDrugs 2020; 1-7. 10.1007/s40259-020-00448-5 33048300 PMC7551520

